# Sustained control of recalcitrant chronic spontaneous urticaria after initiation of inflammatory airway diseases treatment: two case reports

**DOI:** 10.1186/s13256-024-04436-z

**Published:** 2024-02-23

**Authors:** Doanh Nguyen, Philip Deitiker

**Affiliations:** 1 Texas Allergy Group (TAG), Houston, TX USA; 2 Department of Family Medicine, Baylor College of Medicine, Houston, TX USA

**Keywords:** Recalcitrant urticaria, Lower airway disease, Subclinical asthma, Remote site inflammation, Case report

## Abstract

**Background:**

Current classification of chronic urticaria is primarily based on clinical presentation of skin manifestations. Hence, therapeutic treatment is primarily aimed locally for immediate symptom relief. We reason that limiting therapeutic strategies to the skin pathology might be inadequate since cellular activation and inflammation might be triggered remotely.

**Case presentation:**

In this series two patients had exhausted all current treatments for recalcitrant urticaria but remained symptomatic. The first case was 26-year-old Caucasian female and the second was 63-year-old African American female. Both cases had frequent breakthrough urticaria requiring frequent pulsating courses of prednisone to control urticaria despite treatment with omalizumab and antihistamines. When inflammatory airway disease was discovered and managed with inhaled corticosteroid, urticaria is controlled much faster without the need of high dose immunosuppression over several years of observation. Coincidentally, autoimmune thyroiditis and anti-immunogobulin-E immunoglobulin-G titers dropped significantly in one case with sustained inhaled corticosteroid therapy.

**Conclusions:**

We suggest a novel approach of controlling remote epithelial site inflammation in these two cases that resulted in sustained-control of urticaria symptoms without the need for systemic corticosteroids or immunosuppressant. The changes of autoimmune antibodies might be the consequences of tolerance breaking from chronic lower airway inflammation as observed in other epithelial inflammatory condition like in celiac disease and rheumatoid arthritis.

## Background

Chronic Urticaria (CU) can be subdivided into several causal categories ranging from physical (pressure), allergic, hereditary (mastocytosis, hereditary angioedema, and cryopyrin-associated periodic syndromes, and gender), and autoimmune (anti-F_c_ε, anti-IgE and Anti-F_c_εRI autoantibodies, aAbs) with a remnant idiopathic subset [1, 2]. Revealing causal agents in CU is an effective strategy for tailoring treatments. CU classification however is not without problem as certain evident criteria may not be causal. There are several areas of weakness in above categories. In the genetic category familial auto-inflammatory diseases have higher penetrance, but most genetic risk factors have lower penetrance and require other genetic and environmental factors [3, 4]. Autoimmune disease associations (genetic or clinical) with CU are complex [4]. The predominant CU autoimmune risk is characterized by IgE or IgE receptor specific serum IgG. CU aAbs of these types are capable of causing an autologous skin reaction. There are a variety of autoimmune diseases that are found increased in CU (e.g. autoimmune thyroid disease) and CU is increased in autoimmune disease (e.g. celiac disease, CeD). An example of a ‘promising cause’ is systemic lupus erythematosus, which can produces aAbs to IgεR1 receptor, but such aAbs are rarely pathogenic. In addition, thyroid autoimmunity appears to be CU linked with increased risk of severe presentation associated with anti-TPO antibodies [4, 5].

CeD increases risk for multiple inflammatory diseases so the reasoned involvement for CU associations is neither certain or specific. However, some of the involved glutens are indirect tight-junction antagonist (e.g. A9-gliadin via CXCR3 and Zonulin) and innate immune stimulants. Both types of motifs are capable of increasing permeability outside of clinical CeD [6]. Notable with gluten-sensitive patients, treated or untreated, is the sensitivity to other permeability agents, ‘pseudoallergens’ (non-steroidal anti-inflammatory drugs, tartrazine, benzoates, and salicylates), risks shared by CU; although CU has a notably longer list of such allergens [7]. The link between CeD and CU maybe subtle; differential intestinal permeability associated with inflammation may be linked with multiple autoinflammatory diseases (e.g. Type 1 diabetes) whereby permeation precedes the spread of inflammation to other sites.

In recalcitrant CU we reason that more attention on remote epithelial sites of inflammation is needed. First, IgE involvement in CU implicates epithelia involvement but not exclusively skin. Second, urticaria shares many biomarkers with nasal polyps and asthma suggesting that inflammation at other epithelial sites could promote CU [8]. Finally, remote epithelial site inflammation offers more targeted treatment strategies, since all the potential entry points of allergens, sensitizers and infectious agents are epithelial. The epithelia can be roughly subdivided between the skin, the intestinal tract, and the lungs. In addition, experimental studies in mice demonstrate that gut mast cell can be activated remotely by stimulating mast cells in the skin [9]. Logically, the epithelia of airways, skin and lumen could act remotely to promote disease at two major alternative epithelial tissues for 6 total remote-site possibilities. In the case of CU, after excluding by diet the gut-active agents, lower airway inflammation (LAI) acting remotely is the sole remaining possibility. Does undetected LAI qualify as a systemic inflammation and how so? In the following report we present the complex presentation of 2 involved cases of recalcitrant ‘idiopathic’ CU. Lung involvement in both cases went unrecognized and CU treatment improved with LAI testing and treatment.

## Case 1

A 26-year-old Caucasian female with history of lifelong severe atopy, allergic rhinosinusitis, recurrent bronchopneumonia, and recurrent laryngotracheobronchitis. At age 9 she was diagnosed for eosinophilic esophagitis and treated with Fluticasone-HFA swallow until symptoms spontaneously resolved at puberty. Near age 20 there was a period of recurrent bronchitis. She is also a lifelong cat owner with IgE detected to cat dander. There is also a history of reactions to vaccines with dyspnea and urticarial flare up as well as a history of ‘allergic’ responses to multiple medications. There is a family history of asthma.

Two years prior (mid 2019, prior to COVID-19) to being evaluated at TAG the subject developed spontaneous urticaria and angioedema. She was placed on increasing doses of chronic prednisone (4–6 months) with final dose at 300 mg twice daily. The high doses could not be continued due to side-effects (orthopedic and metabolic). Since then, her urticaria symptoms persisted despite high doses of anti-inflammatory agents (fexofenadine, 720 mg/day; montelukast; doxepin 100 mg/day; and Omalizumab 300 mg/14 day). In addition, frequent flushing required intramuscular self-injected diphenhydramine. A year prior the patient was also treated for SARS-CoV-2 infection requiring hospitalization.

**Fig. 1:**
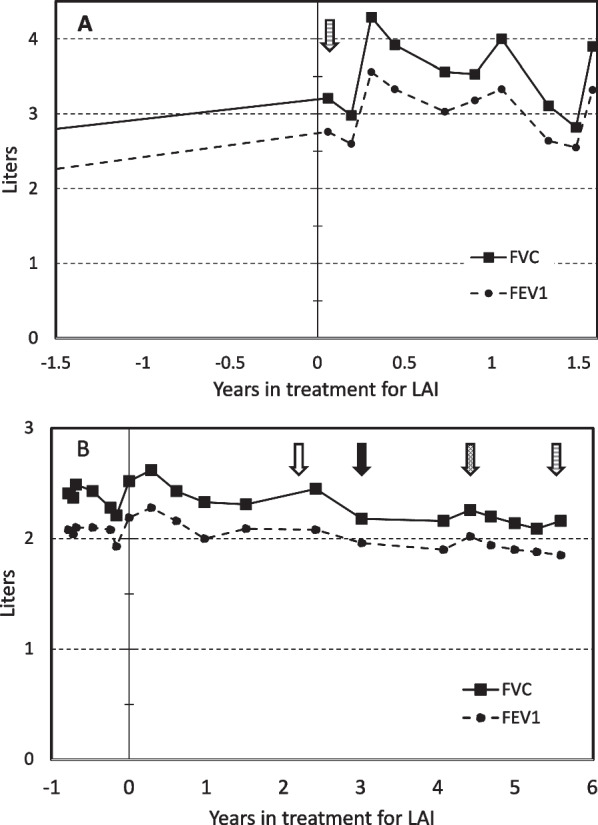
Pulmonary function tests for Case 1 (**A**) and Case 2 (**B**). The solid line shows forced vital capacity and the dashed line shows forced expiratory volume (1 s) for both patients. The striped arrow in **A** represents the treatment-initiation of Dupilumab 300 mg SC. The open arrow in **B** represents the approximate time patient elected to discontinue treatment, filled arrow represents when treatment was restarted with budesonide/formoterol 160 mcg/4.5 mcg aerosol, the pixelated arrow represents the increase in Omalizumab updosing to 375 mg, the striped arrow represents the treatment-initiation of Dupilumab 300 mg SC in addition to Omalizumab

On initial evaluation at TAG the patient exhibited discomfort, with observed skin flushing and self-injection as described. Patient presented with severe allergies including erythematous bilateral tympanic membranes, erythematous nasal and throat mucosa, but with clear lungs and no wheezes or rales. Notable indicators of LAI included: dyspnea on exertion or wearing facemask, chronic fatigue and sleepiness. Spirometry done two years prior shows Forced Vital Capacity (FVC) at 66% and Force Expiratory Volume at 1 s (FEV1) at 60% of the predicted values. Other inflammatory symptoms include: abdominal bloating, and diarrhea. Prior to treatment with Omalizumab (anti-IgE) total IgE was in 400 U/ml. Labs evaluation demonstrated positive anti-thyroglobulin 28 IU/ml, positive anti-IgE IgG with titer of 42 μg/ml, tryptase 3.8 μg/L, eosinophil 60 cells/μl, total IgE of 415 U/ml with detectable sIgEs to cockroach, dust mites (2 species) and cat dander (Quest Diagnostics). Spirometry evaluation showed a persistent moderate restriction and obstruction (Fig. 1a; Table 1). This case’s clinical presentation is consistent with chronic spontaneous urticaria with transient lesions typical of idiopathic urticaria including erythema and blanching. Such urticarial lesions are atypical of vasculitis, which is not transient and results in darkened macules. Patient did not present with bullae, purpura, edematous patches, necrotic lesions or differential distribution of erythema to sun-light exposed areas.

**Table 1:** Temporal immune and urticaria profiles for case 1

	Year (after LAI diagnosis)	
	0.0	1.3
Cellular findings		
Eosinophils	60	–
Antibody findings		
Ȃ-Thyroglobulin	28 (28X)	3 (3X)
Ȃ-Thyroid peroxidase	1	1
Ȃ-IgE	42 (250X)	2 (13X)

*Ȃ-IgE* Anti-Immunoglobulin E, are given in mg/ml and for other autoimmune responses Ȃ is Antibody against given self-protein, in international units. The variable values of autoantibodies to IgE may reflect the treatment of Omalizumab and the patients anti-idiotypic response to the same medication. X values are given as the test value divided by disease threshold values for the test

Due to indicators of LAI the patient was promptly started on Budesonide plus albuterol sulfate nebulizer with an instruction to start with partial treatment of a vial and updosing as tolerated. With a suspicion of excessive microbial colonization of the lower airways, the patient was started on azithromycin for 5 days. A week later, the patient reported a significantly lower frequency of facial flushing and hives, and she also noticed that breathing was easier. Patient was given loading doses of Dupilumab (300 mg over 2 days). At subsequence visits, the patient continued to experience some intermittent bronchospasm and hives but significantly less frequent. After tolerating Budesonide nebulizer, the inhalant was updosed to Budesonide/Formoterol HFA 160/4.5 (aerospacer with mask) 2 puffs at twice a day.

The patient continues to do well without significant hives flare up with controlled exposure to pets and additional domestic air filtration (Table 1; Fig. 1a). Due to drug-induced hip injury, the patient required remedial surgery, which resulted in a transient flare up in urticarial and LAI, controlled without the use of high-dose corticosteroid. The presence of IgE Autoab also dropped during the course of treatment from 42 to 2 µg/ml (Table 1) over the course of treatment. The thyroxine autoantibodies dropped from 28 to 3 IU/ml. During the spring and fall some symptoms would return with loss of lung volume. To control episodic responses, she was placed on extra cromolyn and a second inhaled corticosteroid during periods of higher pollen. There was one minor flare up during the 2nd year of treatment due to a malfunctioning domestic air-handling system.

## Case 2

A 63-year-old African American female with history of lifelong allergic rhinoconjunctivitis, recurrent bronchitis and wheezing came for evaluation of one year history of uncontrolled recalcitrant urticaria. The patient also has diabetes and breast cancer 4 years prior status post left-mastectomy and chemotherapy. Labs at symptom onset showed basophil activity 501% (Clinical Pathology Laboratories; normal ≤ 27%)**.** Urticaria symptom remained uncontrolled on Omalizumab 300 mg/4 weeks for the previous year, fexofenadine 180 mg/day, famotidine, hydroxyzine and prednisone 10 mg (2 ×)/day.

Initial examination at TAG showed diffuse hives with urticaria (QoLS 85), rhinitis (QoLS 45). This case’s clinical presentation is consistent chronic spontaneous urticaria with transient lesions typical of idiopathic urticaria including erythema and blanching. Urticarial vasculitis was ruled out based on nop clinical presentation of darkened macules when hives resolve and lack of vasculitis markers (Table 2). The patient did not present with bullae, purpura edematous patches, necrotic or differential erythema to sun-exposed areas. Spirometry demonstrated a low peak flow, moderate restriction and a significant reversibility of FEF25-75 after bronchodilator challenge. Initial labs while on Omalizumab and oral prednisone showed total IgE of 258 U/ml, eosinophil 0 cells/μl, chronic urticaria index (second generation Functional Anti-FceR test—Labcorp) of > 50.0% with normal value < 10%. Once off prednisone for more than 4 weeks**,** subsequent lab work revealed a total IgE of 171 U/ml, eosinophil 213 cells/μl with detectable sIgE to cockroach and both dust mites species. Hives were controlled (QoLS 32) after one week, and she was placed on beclomethasone dipropionate HFA 80 mcg/puff.

**Table 2:** Temporal immune and urticaria profiles for Case 2

	Year (after LAI diagnosis)				
	− 1.2	0.1	4	5.2	6.2
Cellular findings					
Eosinophils	100	200	100	300	
Complement (mg/dL)					
C1Q					7.8 (0.9X)
C3					203 (1.03X)
C4					28 (0.5X)
Antibody findings					
IgE		258		2218	
Ȃ-Thyroglobulin	< 1	< 1		< 1	
Ȃ-Thyroid Peroxidase	0.22 ×			1 ×	
Ȃ-C1q					Negative
Ȃ-IgεR1		50 CUI (5X)			
Ȃ-Igε				64 (328X)	
BAT	501 (27X)				
ANCA					Negative
Ȃ-Myeloperoxidase					< 1
Ȃ-Proteinase 3					< 1

Ȃ is Antibody (Ab) against given self-protein. BAT is a Basophil activation test and was data obtained from another site prior to treatment. The high values of autoantibodies to immunoglobulin E (IgE), given in mg/ml, may reflect the treatment of Omalizumab, a humanized monoclonal antibody to IgE. The Ȃ-IgεR1 IgE receptor test was unavailable in 2022. Patients elected to stop inhaler treatment before year 4 and resumed treatment between Year 4 at Year 5.2. ANCA is Anti-neutrophilic cytoplasmic antibodies. X values are given as the test value divided by disease threshold values for the test for elevated results

While continuing use of Omalizumab the following treatments were added: inhaled beclomethasone—dipropionate HFA (80 μg; 2 puffs twice daily), doxepin (25 mg, nightly) and hydroxychoroquine (200 mg, daily). However the initial 60 mg dose of prednisone daily was gradually eliminated over one month.

Over the next 5 years, the patient continued to do well without major urticarial flare up or the need for systemic corticosteroid use. The patient elected to cease inhaler use after 18 months. However, in the fifth-year patient developed sensations of itchiness in the palms and feet without hives. Lung function test also showed a loss of volumes faster than predicted. Therefore, the patient was restarted on an ICS/LABA (budesonide/formoterol 160/4.5) inhaler plus doxepin 50 mg daily. Lung function test reveal a decline of lung volumes over 5 years. While initial anti-IgE was not established the test performed indicated both AutoAb to IgE and its’ receptor. The most recent test for anti-IgE was 64,246 ng/ml and may reflect recent treatment with Omalizumab. Thyroglobulin AutoAb exceeded the normal range but remained constant; and thyroxine peroxidase AutoAbs appear to have risen fourfold. We recently looked at some common autoimmune markers (anti-C1Q, anti-neutrophil cytoplasmic antibodies, anti-myeloperoxidase antibodies, antiproteinase 3 antibodies) for vasculitis; however, none of the markers were positive for occult autoimmune disease (Table 2). Moreover we also evaluated complement components C1q, C3 and C4; however, only C3 deviated from normal range (Table 2, 3% outside range), whereas in hypocomplementemic urticarial vasculitis (McDuffie syndrome) these markers are expected lower.

## Discussion

In these two case studies patients underwent lengthy treatment for recalcitrant chronic urticaria with unsatisfactory results. In the first case study anti-inflammatory treatment peaked with 600 mg of systemic corticosteroid per day, eventually leading to deleterious and permanent side-effects. The introduction of inhaled anti-asthmatic drugs reduced many of the symptoms and facilitated the treatment of the remaining symptoms. Despite indicators of autoimmune disease involvement, the detection of remote LAI appeared to best predict treatment strategy. Had asthmatic inflammation been detected earlier better treatment strategies would have been available for detection and remediation of causal environmental antigens. With control of the LAI the other indicators of autoimmune disease decreased.

For both cases, improvement of urticarial symptoms were observed soon after initiation of inhaled corticosteroid for treatment of underlying LAI. Both cases were placed on much higher doses of systemic corticosteroid without symptom control. Plasma concentration of inhaled corticosteroid would be significantly lower than systemic corticosteroid treatment that the subjects were placed on previously [10]. This would argue against the direct effect of inhaled corticosteroid in controlling CU via direct systemic effects since previously much higher plasma concentrations of systemic corticosteroids were tried and found ineffective. The improvement and resolution of urticarial lesions on the skin would also argue against systemic effect of inhaled corticosteroid on lesions if those were of autoimmune induced vasculitis origins. In other words, the plausible explanation of this inhaled corticosteroid effect is by reducing LAI leading to improvement and eventual resolvement of remote mast cell stimulation in the skin or remote epithelia.

In the first case, symptoms appeared prior to SARS2 pandemia and the relatively consistent presentation before and after infection suggests COVID-19 related vasculitis is not causal. The lack of clinical features for bullous pemphigoid (bullae), Henoch-Schönlein (purpura and remnant discoloration), IgM/IgG immune complex vasculitis (purpura), tumid lupus erythematosus (differential distribution of lesions to sun exposed areas), Well’s syndrome (edematous patches), erythema multiforme (bullous or necrotic lesions), cutaneous mastocytosis (macules or papules), cryopyrin-associated periodic syndrome (cold urticaria, fever) further limit diagnosis to idiopathic urticaria. In addition the inhaled corticosteroids used to treat this patient would have no appreciable effect on bullous pemphigoid, Henoch-Schönlein purpura, IgM/IgG immune complex vasculitis, tumid lupus erythematosus, Well’s syndrome, erythema multiforme or cryopyrin-associated periodic syndrome. The inhaled corticosteroids might be effective in some cutaneous mastocytosis patients, with the systemic corticosteroids more so, but systemic corticosteroids were ineffective in treatment at early stages of disease. It should be pointed out that in anti-IgE testing we speculate that testing may not discriminate some humanized monoclonal antibodies (Omalizumab) from endogenous autoAb to IgE. The drop in anti-IgE levels on second testing may reflect immune responses to the monoclonal antibodies.

In the second case there was upper airway inflammation but no obvious signs of LAI despite a remote history of recurrent bronchitis. The involvement of LAI was obscured by race-dependent lung volume and air-displacement standards likely leading to missed diagnosis at presentation. When using Caucasian predicted values, tests for flow restriction indicated the patient lung performance fell below expectations. Lung function tests showed restriction plus significant reversibility of early obstruction with bronchodilator indicating already developed LAI. As a consequence, the patient was treated for active LAI and this treatment improved lung performance and skin pathology. In this case the patient was not convinced to continue inhaler usage (unfilled arrow, Fig. 1b). The patient later relapsed and continued LAI treatment (filled arrow, Fig. 1b). The indicators of autoimmune disease did not decrease, and may have increased. A concern developed whether this could be urticarial vasculitis with pulmonary involvement; however, the autoimmune makers and urticarial vasculitis symptoms are not present (rapidly onset, with destruction and necrosis of pulmonary tissue). Like case 1, case 2 did not present with visual features of bullous pemphigoid, Henoch-Schönlein purpura, IgM/IgG immune complex vasculitis, tumid lupus erythematosus, Well’s syndrome, erythema multiforme, or cutaneous mastocytosis, and lacked fever-associated with cryopyrin-associated periodic syndrome.

The role of systemic tissue damage in chronic epithelial inflammation deserves some discussion. It is known from the study of autoimmune disease that once autoimmune status becomes positive, that status is irreversible despite some effective treatments. Moreover, the risk of a nascent second autoimmune disease increases with that status. This trend is evident in recalcitrant urticarial with IgE autoimmunity, which increases risk for autoimmune thyroid disease, or potentially, vice versa. Strategically, it is desirable to intervene in disease causality before a causal inflammation such as LAI becomes refractory or permanent damage occurs (either by disease or its treatment). And the question in LAI concerns disease development through the various involved tissues and the mechanism of remote transfer. The fact that these two patients both show evidence of autoimmune diseases (2) is indicative of a persistent inflammatory environment, and the untreated nature of the LAI is a potent candidate for the initial site of inflammation. In the first case the continued treatment of the LAI was followed by a reduction of aAb to IgE. In the second case treatment with inhaler was ceased after a brief period of treatment and symptoms returned along with an elevated anti-IgE level. In this second case the thyroglobulin aAbs remained high and thyroxine peroxidase aAbs rose.

The association of nascent autoimmune disease with preexisting autoimmune disease has been established. A good model is Celiac Disease. But it has been noted that in Celiac Disease, cohorts of at-risk patients that show Marsh grade 1 or 2 gluten-sensitive enteropathy have increased risk for other autoimmune diseases. This indicates that a formative environment can exist prior to the presence of autoantibody markers for disease. With gluten sensitivity there are a number of secondary autoimmune diseases associated as part of poly-autoimmune disease syndromes (Type 1 diabetes, Addison’s disease, Autoimmune thyroiditis, Autoimmune hepatic disease) and a whole spectrum of autoimmune diseases that have slightly increased risk. Inflammation within the lamina propria (early) and the basal lamina (clinical disease) provide an environment where luminal antigens can more readily infiltrate the lamina propria and cause occult inflammation. The examples here present a similar situation but with a general lack of understanding the nature of the underlying tissue inflammation. In Case 1 there was a suspicion of a prolonged increase of microbial colonization that was treated early in disease. The presence of chronic inflammation caused by the accumulation of microbial antigens may have created a tolerance breaking circumstance via epitope evolution and antigen mimicry [11, 12]. Alternatively, the inflammation may have caused the accumulation of B and T lymphocytes resulting in remodeling. In the second case, due to the lack of evidence for excessive colonization and generally marginal indications of autoimmune thyroid disease, antigen mimicry is less likely. But, the modest rise of a second autoimmune antibody marker and the generally high current level of anti-IgE after a prolonged lapse of LAI treatment suggests that chronic untreated inflammation could be primary cause of autoimmunity.

Another concern arises from these two cases; the criteria for qualifying asthma and misdiagnosis. Unlike ‘canonical’ asthma, LAI involved in CU is occult, consequently more objective measurement criteria are needed. The patient may elect to discontinue treatment without realizing that inflammation might reoccur once the inhalers are discontinued, inflammation may be difficult to initially detect. In the aftermath of pandemia of nascent human respiratory viral infection of chronic LAI maybe lumped into general categories such as “long covid”. This may be part of sequela of lower airway infection with both prolonged inflammation and/or opportunistic colonization as autoinflammatory diseases mediators. These two possibilities indicate the importance of better testing, particularly in the case of excessive colonization.

An additional concern involves race-based lung statistics that may not fairly represent normal subpopulations, resulting in under-diagnosis and excessively long periods of chronic inflammation. In the case of African Americans, individuals come from many parts of Africa and have a spectrum of ancestors from Europe, North America, or Asia; consequently, the statistics may mask LAI.

## Conclusion

These two cases present both similarity and differences in the relationship between LAI and CU. The similarities demonstrate both cases have underdiagnosed LAI, IgE aAbs, and the presence of markers for unrelated autoimmune disease. One case shows a microbial involvement potential. One case demonstrates a decline of aAb markers with continued treatment and the other show either stasis or increase of aAb markers with a discontinuous treatment. The autoantibodies appear to be associated with, but not necessarily causal of, CU in both cases. The treatment with inhaler and modulation of airborne sensitizers appears to be key to the reduction in CU. The actual aAb markers may be less critical in CU status, while the persistent autoinflammatory environment promoted by occult LAI spreads systemically in this variation of ‘recalcitrant’ urticaria. Strategically the link between LAI and CU improves treatment and diagnostic strategies for occult asthma and excessive airway colonization.

## Data Availability

Additional data are available from the corresponding author upon reasonable request.

## Abbreviations

aAb: Autoimmune antibody
CU: Chronic Urticaria
IgE: Immunoglobulin E
LAI: Lower airway inflammation
FVC: Forced volume capacity
FEV1: Forced effective volume (1 s)
FEF25-75: Forced expiratory flow 25–75%
QoLS: Quality of life score
